# Pre-treatment GlycA measurement provides no additional predictive utility beyond routine clinical measures in patients with rheumatoid arthritis

**DOI:** 10.1093/rheumatology/keaf235

**Published:** 2025-05-13

**Authors:** Stephanie F Ling, Chuan Fu Yap, Nisha Nair, Suzanne M M Verstappen, Ann W Morgan, John D Isaacs, Anthony G Wilson, Kimme L Hyrich, Anne Barton, Darren Plant

**Affiliations:** Centre for Genetics and Genomics Versus Arthritis, Centre for Musculoskeletal Research, The University of Manchester, Manchester, UK; NIHR Manchester Biomedical Research Centre, Manchester University NHS Foundation Trust, Manchester Academic Health Science Centre, Manchester, UK; Centre for Genetics and Genomics Versus Arthritis, Centre for Musculoskeletal Research, The University of Manchester, Manchester, UK; Centre for Genetics and Genomics Versus Arthritis, Centre for Musculoskeletal Research, The University of Manchester, Manchester, UK; NIHR Manchester Biomedical Research Centre, Manchester University NHS Foundation Trust, Manchester Academic Health Science Centre, Manchester, UK; NIHR Manchester Biomedical Research Centre, Manchester University NHS Foundation Trust, Manchester Academic Health Science Centre, Manchester, UK; Centre for Epidemiology Versus Arthritis, Centre for Musculoskeletal Research, The University of Manchester, Manchester, UK; School of Medicine, University of Leeds, Leeds, UK; NIHR Leeds Biomedical Research Centre, Leeds Teaching Hospitals NHS Trust, Leeds, UK; Translational and Clinical Research Institute, Newcastle University, Newcastle-upon-Tyne, UK; Musculoskeletal Unit, Newcastle-upon-Tyne Hospitals NHS Foundation Trust, Newcastle-upon-Tyne, UK; School of Medicine and Medical Science, Conway Institute, University College Dublin, Dublin, Ireland; NIHR Manchester Biomedical Research Centre, Manchester University NHS Foundation Trust, Manchester Academic Health Science Centre, Manchester, UK; Centre for Epidemiology Versus Arthritis, Centre for Musculoskeletal Research, The University of Manchester, Manchester, UK; Centre for Genetics and Genomics Versus Arthritis, Centre for Musculoskeletal Research, The University of Manchester, Manchester, UK; NIHR Manchester Biomedical Research Centre, Manchester University NHS Foundation Trust, Manchester Academic Health Science Centre, Manchester, UK; Centre for Genetics and Genomics Versus Arthritis, Centre for Musculoskeletal Research, The University of Manchester, Manchester, UK; NIHR Manchester Biomedical Research Centre, Manchester University NHS Foundation Trust, Manchester Academic Health Science Centre, Manchester, UK

Rheumatology key message

- Pre-treatment measurement of the inflammatory biomarker GlycA is unlikely to provide predictive utility of treatment response in patients with RA starting either adalimumab or methotrexate.

Dear Editor, Due to its status as a complex heterogeneous disorder and further heterogeneity in its treatment outcome measures, RA still has few validated pre-treatment biomarkers of response to therapy [1]. GlycA was originally identified as a composite NMR signal representing mostly *N*-acetyl glucosamine residues on the carbohydrate side chains of a number of acute-phase reactants, including α_1_-antitrypsin and haptoglobin [2]. Previous studies have reported associations between GlycA and DAS28-ESR [3] and baseline GlycA measurement and DAS28 remission after 6 months of treatment with conventional synthetic (cs) DMARD therapy [4]. We sought to validate these associations in two independent cohorts of patients with RA, one with early disease starting methotrexate, and the other with established disease, starting adalimumab.

Patients with early RA participating in the RA Medication Study (RAMS) were included, comprising patients aged ≥18 years with a physician diagnosis of either RA or undifferentiated inflammatory polyarthritis who were starting methotrexate for the first time [5]. Patients with established RA commencing adalimumab (including biosimilars) were included from the Biologics in RA Genetics and Genomics Study Syndicate (BRAGGSS), comprising patients aged ≥18 years fulfilling the 1987 ACR classification criteria for RA [6]. Healthy controls (HCs) were selected from the National Repository Study, comprising healthy volunteers. In all RA participants, clinical data were obtained. DAS28-CRP (two-, three- and four-component algorithms, 2C, 3C and 4C, respectively) and Clinical Disease Activity Index (CDAI) were calculated (see Supplementary Methods, available at *Rheumatology* online). Missing disease activity measure sub-components (Supplementary Table S1, available at *Rheumatology* online) were imputed using a random forest algorithm [7]. Serum samples were obtained from all RA cases at baseline (pre-treatment) and from HCs at a single time-point. Repeat samples were taken from the RAMS patients after 4 weeks of treatment and from the BRAGGSS patients after 3 months. Sera were sent to Nightingale Health Plc (Helsinki, Finland) for NMR spectroscopy to quantify GlycA levels; data were normalized using probabilistic quotient normalization and scaled using pareto-scaling.

All statistical analysis was carried out using R v.4.4.1 [8]. GlycA values were non-parametrically distributed. The Mann–Whitney *U* test was used to compare distributions between cases and HCs, treatment groups, responders and non-responders and patients in remission and HCs. Logistic regression was used to explore relationships between GlycA and treatment response categories, adjusting for age, sex, drug and pre-treatment clinical outcome measures (where appropriate). Correlations between GlycA expression values and continuous clinical outcome measures after 3 months were calculated using Pearson’s correlation coefficient.

In total, 199 patients with RA were included (99 adalimumab, 100 methotrexate) and 49 HCs. Median age in RA was 61.16 years (interquartile range, IQR, 53.33–68.84), with 166 female patients (83.42%). There were 32 female HCs (65.31%), with a median age of 46 years (IQR 37–55). Full summary statistics are listed in Supplementary Table S2, available at *Rheumatology* online. In the combined RA group, GlycA values were significantly higher than HC values at both pre-treatment and follow-up (Supplementary Table S3, available at *Rheumatology* online). In all RA patients, regardless of drug received, pre-treatment GlycA levels were significantly associated with pre-treatment 2C/3C/4C-DAS28-CRP, but not CDAI (Supplementary Tables S4–S9, available at *Rheumatology* online).

Pre-treatment GlycA values were not associated with treatment response at 3 months measured using 2C/3C/4C-DAS28-CRP and CDAI, following adjustment (Supplementary Tables S10–S15, available at *Rheumatology* online). RA patients in remission (2C/3C/4C-DAS28-CRP <2.6, CDAI <2.8) did not have significantly different GlycA distributions compared with HCs (Supplementary Table S16, available at *Rheumatology* online). Conversely, patients with active disease (2C/3C/4C-DAS28-CRP ≥2.6, CDAI ≥2.8) had significantly different distributions (Supplementary Table S17, available at *Rheumatology* online), implying that patients in disease remission had GlycA values more similar to health. The only disease activity measure sub-component significantly correlated with baseline GlycA at follow-up was CRP in the combined RA group (*r* = 0.27, *P* = 3.09E−04) and the adalimumab-only subgroup (*r* = 0.36, *P* = 9.00E−04), (Supplementary Tables S18–S20, available at *Rheumatology* online, Fig. 1). There were no correlations between change in GlycA between baseline and (i) 3 months in adalimumab and (ii) 4 weeks in methotrexate, or change in clinical outcome measures between baseline and 3 months (Supplementary Tables S21–S23, available at *Rheumatology* online).

**Figure 1.**
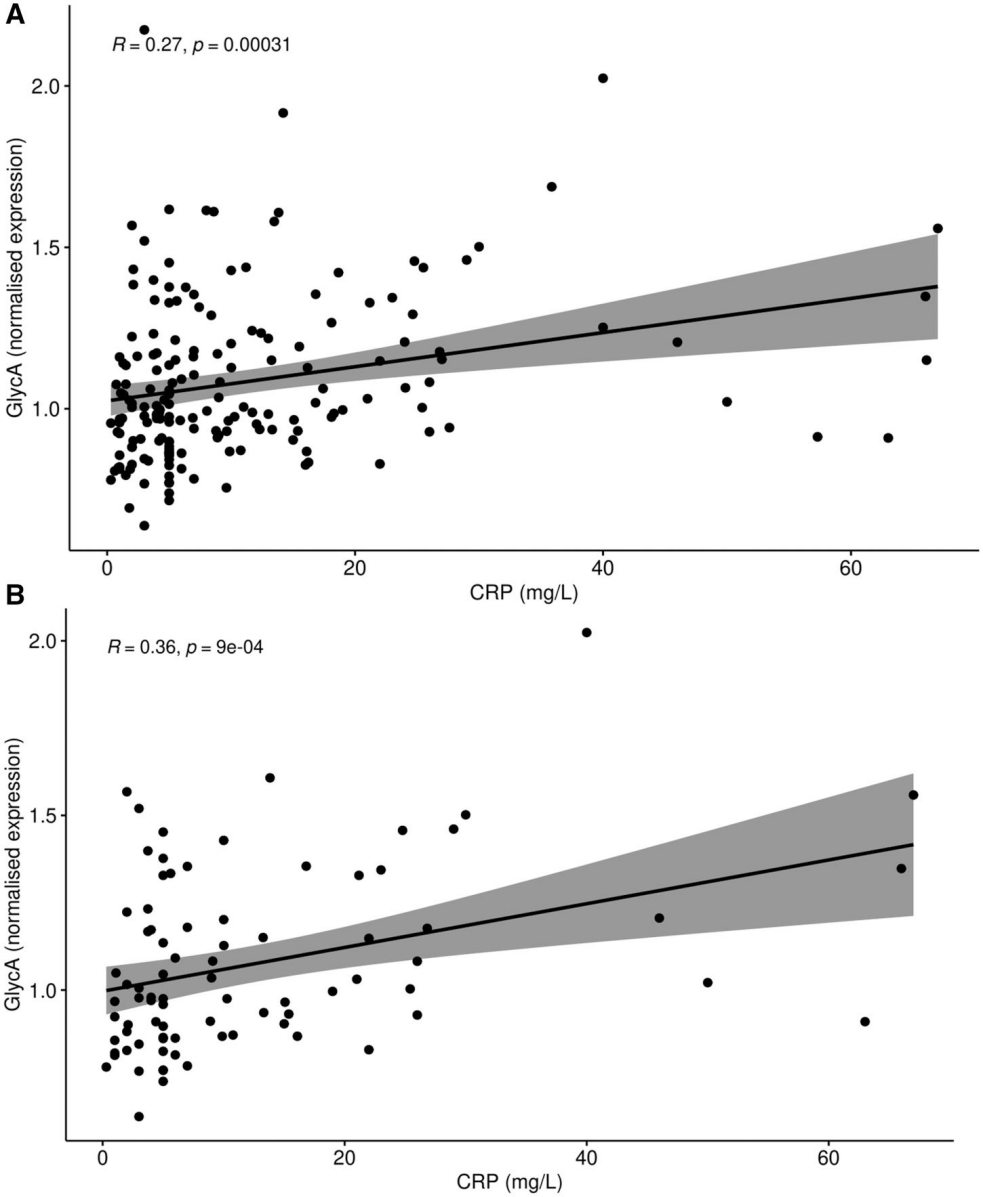
Correlation between serum CRP levels after 3 months of treatment and pre-treatment GlycA. (A) Both adalimumab and methotrexate patients combined. (B) Adalimumab patients only

Our findings suggest that GlycA as a pre-treatment biomarker of future response in patients with RA is unlikely to add any additional information over clinical measures that are currently in use, particularly given its strong correlation with CRP. The strongest associations were between pre-treatment GlycA and CRP at 3 months in patients treated with adalimumab; this relationship was absent in patients treated with methotrexate. This could be due to a drug- or disease stage-specific effect (i.e. early vs. established RA). Our findings agree with Bartlett *et al*. [3], but not those of Rodriguez-Carrio *et al*. [4]; in the latter study, clinical outcomes were assessed at 6 months using DAS28-ESR, so differences in study design may explain the conflicting findings. In conclusion, we found that GlycA adds no additional clinical information beyond usual CRP measurement in the current study.

## Supplementary Material

keaf235_Supplementary_Data

## Data Availability

Data are available on request to the corresponding author; data requests are at the discretion of the corresponding author.
