# Supplementary material for: Pre-treatment GlycA measurement provides no additional predictive utility beyond routine clinical measures in patients with rheumatoid arthritis
Source: Rheumatology (Oxford). 2025 May 13;64(7):4417–9. doi: 10.1093/rheumatology/keaf235 (PMC12212900; doi:10.1093/rheumatology/keaf235)
Supplement: keaf235_Supplementary_Data [file keaf235_supplementary_data.docx]

**SUPPLEMENTARY METHODS**

*Calculation of two-component Disease Activity Score of 28 Joints (2C-DAS28)*

$$2CDAS28CRP=\sqrt{SJC28}+\left( 0.6 x ln\left( CRP+1 \right) \right)$$

As per Hensor *et al*^1^, where CRP = C-reactive protein and SJC = swollen joint count.

*Calculation of three-component DAS28 (3C-DAS28)*

$$3CDAS28CRP= \left[ \left( 0.56 \times\surd TJC28 \right)+\left( 0.28\times\surd SJC28 \right)+\left( 0.36 \times ln\left( CRP+1 \right) \right) \right]\times1.10+1.15$$

As per DAS-Score website^2^, where TJC = tender joint count.

*Calculation of four-component DAS28 (4C-DAS28-CRP)*

$$4CDAS8CRP=\left( 0.56 \times\surd TJC28 \right)+\left( 0.28\times\surd SJC28 \right)+ \left( 0.36\times ln\left( CRP+1 \right) \right)+\left( 0.014 \times GHVAS \right)$$

As per Prevoo *et al*^3^, where GHVAS = patient global health visual analogue scale (0 – 100 mm).

*Calculation of Clinical Disease Activity Index (CDAI)*

$$CDAI=SJC+TJC+PGA+EGA$$

As per Aletaha *et al*^4^, where PGA = patient global assessment of disease activity (0 – 10 cm) and EGA = evaluator global assessment of disease activity (0 – 10 cm).

*Calculation of EULAR response*

| **DAS28 at endpoint** | **Improvement in DAS28 from baseline** | | |
| --- | --- | --- | --- |
|  | ≥1.2 | >0.6 and ≤1.2 | ≤0.6 |
| ≤3.2 | Good |  |  |
| >3.2 and ≤5.1 |  | Moderate |  |
| >5.1 |  |  | None |

As per van Gestel *et al*^5^.

*Calculation of CDAI response*

| **CDAI at endpoint** | **Response category** |
| --- | --- |
| <2.8 | Remission |
| 2.8 – 10 | Low |
| 10.1 – 22 | Moderate |
| >22 | High |

As per Aletaha *et al^6^*.

**SUPPLEMENTARY RESULTS**

Supplementary Table S1. Number of missing disease activity scoring sub-components.

|  | **Adalimumab baseline (n = 97)** | **Adalimumab 3 months (n = 84)** | **Methotrexate baseline (n = 100)** | **Methotrexate 3 months (n = 98)** |
| --- | --- | --- | --- | --- |
| TJC | 2 | 2 | 0 | 3 |
| SJC | 2 | 2 | 0 | 3 |
| Patient GH | 1 | 6 | 0 | 5 |
| Physician GH | 23 | 19 | 0 | 1 |
| CRP | 21 | 21 | 0 | 10 |

**Abbreviations:** CRP (C-reactive protein), GH (global health), SJC (swollen joint count), TJC (tender joint count).

Supplementary Table S2. Baseline cohort characteristics.

| **Characteristic** | **All RA (n = 199)** | **Adalimumab only (n = 99)** | **Methotrexate only (n = 100)** | **HCs (n = 49)** | **p-value (all RA vs HCs)** |
| --- | --- | --- | --- | --- | --- |
| Age (years), median [IQR] | 61.16 [53.33, 68.84] | 59.55 [50.55, 66.00] | 62.53 [55.80, 71.92] | 46 [37, 55] | 4.76E-11 |
| Female sex, n (%) | 166 (83.42) | 87 (87.88) | 79 (79.00) | 31 (65.96)  [2 missing] | 4.93E-27 |
| Pre-treatment DAS28-CRP(4C), median [IQR] | 5.35 [4.57, 5.99] | 5.66 [5.25, 6.25] | 4.69 [4.07, 5.68] | N/A | N/A |
| Pre-treatment DAS28-CRP(3C), median [IQR] | 4.92 [4.35, 5.66] | 5.32 [4.72, 5.77] | 4.51 [3.86, 5.39] | N/A | N/A |
| Pre-treatment DAS28-CRP(2C), median [IQR] | 4.25 [3.38, 4.89] | 4.40 [3.81, 4.87] | 3.91 [3.26, 4.89] | N/A | N/A |
| Pre-treatment CDAI, median [IQR] | 32.00 [22.00, 39.00] | 34.90 [29.62, 40.80] | 24.40 [16.85, 63.90] | N/A | N/A |

**Abbreviations:** 2C (two-component), 3C (three-component), 4C (four-component), CDAI (Clinical Disease Activity Index), DAS28-CRP (Disease Activity Score of 28 Joints with CRP), HC (healthy controls), IQR (interquartile range), N/A (not applicable), RA (rheumatoid arthritis).

Supplementary Table S3. Case-control analysis between patients with RA and HCs, using a Mann-Whitney U test to compare groups.

| **Comparison** | **W** | **p-value** |
| --- | --- | --- |
| Both drugs, baseline | 1717 | 2.599E-12* |
| Adalimumab, baseline | 894 | 4.427E-10* |
| Methotrexate, baseline | 823 | 6.689E-11* |
| Both drugs, follow-up | 2961 | 2.647E-04* |
| Adalimumab, follow-up | 1581 | 0.0264* |
| Methotrexate, follow-up | 1380 | 2.063E-05* |

**Abbreviations:** HC (healthy control), RA (rheumatoid arthritis).

Supplementary Table S4. Association of baseline GlycA values with baseline (pre-treatment) disease activity, both drugs combined, univariable analysis.

| **Outcome measure** | **β-coefficient (95% CI)** | **p-value** | **n** |
| --- | --- | --- | --- |
| 4C-DAS28-CRP | 0.99 (0.44 – 1.53) | 5.00E-04 | 198 |
| 3C-DAS28-CRP | 0.9 (0.47 – 1.46) | 1.90E-04 | 198 |
| 2C-DAS28-CRP | 1.87 (1.30 – 2.44) | 9.33E-10 | 198 |
| CDAI | 3.05 (-3.72 – 9.81) | 0.378 | 198 |

**Abbreviations:** 2C (two-component), 3C (three-component), 4C (four-component), CDAI (Clinical Disease Activity Score), CI (confidence interval), DAS28-CRP (Disease Activity Score of 28 Joints with CRP).

Supplementary Table S5. Association of baseline GlycA values with baseline (pre-treatment) disease activity, both drugs combined, adjusted for age at recruitment, sex and drug.

| **Outcome measure** | **β-coefficient_adj_ (95% CI)** | **p-value** | **n** |
| --- | --- | --- | --- |
| 4C-DAS28-CRP | 1.04 (0.55 – 1.52) | 4.19E-05 | 198 |
| 3C-DAS28-CRP | 0.99 (0.52 – 1.47) | 5.84E-05 | 198 |
| 2C-DAS28-CRP | 1.85 (1.28 – 2.41) | 1.34E-09 | 198 |
| CDAI | 3.82 (-2.49 – 10.12) | 0.24 | 198 |

**Abbreviations:** 2C (two-component), 3C (three-component), 4C (four-component), CDAI (Clinical Disease Activity Score), CI (confidence interval), DAS28-CRP (Disease Activity Score of 28 Joints with CRP).

Supplementary Table S6. Association of baseline GlycA values with baseline (pre-treatment) disease activity, adalimumab only, univariable analysis.

| **Outcome measure** | **β-coefficient (95% CI)** | **p-value** | **n** |
| --- | --- | --- | --- |
| 4C-DAS28-CRP | 0.80 (0.20 – 1.39) | 0.01 | 99 |
| 3C-DAS28-CRP | 0.82 (0.25 – 1.39) | 0.01 | 99 |
| 2C-DAS28-CRP | 1.72 (1.02 – 2.42) | 5.58E-06 | 99 |
| CDAI | 2.67 (-5.19 – 10.53) | 0.51 | 99 |

**Abbreviations:** 2C (two-component), 3C (three-component), 4C (four-component), CDAI (Clinical Disease Activity Score), CI (confidence interval), DAS28-CRP (Disease Activity Score of 28 Joints with CRP).

Supplementary Table S7. Association of baseline GlycA values with baseline (pre-treatment) disease activity, adalimumab only, adjusted for age at recruitment and sex.

| **Outcome measure** | **β-coefficient_adj_ (95% CI)** | **p-value** | **n** |
| --- | --- | --- | --- |
| 4C-DAS28-CRP | 0.82 (0.22 – 1.41) | 0.01 | 99 |
| 3C-DAS28-CRP | 0.84 (0.26 – 1.42) | 0.01 | 99 |
| 2C-DAS28-CRP | 1.72 (1.02 – 2.41) | 5.43E-06 | 99 |
| CDAI | 2.97 (-4.99 – 10.94) | 0.47 | 99 |

**Abbreviations:** 2C (two-component), 3C (three-component), 4C (four-component), CDAI (Clinical Disease Activity Score), CI (confidence interval), DAS28-CRP (Disease Activity Score of 28 Joints with CRP).

Supplementary Table S8. Association of baseline GlycA values with baseline (pre-treatment) disease activity, methotrexate only, univariable analysis.

| **Outcome measure** | **β-coefficient (95% CI)** | **p-value** | **n** |
| --- | --- | --- | --- |
| 4C-DAS28-CRP | 1.21 (0.44 – 1.97) | 1.49E-12 | 99 |
| 3C-DAS28-CRP | 1.13 (0.38 – 1.87) | 3.70E-03 | 99 |
| 2C-DAS28-CRP | 2.03 (1.14 – 2.91) | 1.95E-05 | 99 |
| CDAI | 3.87 (-6.01 – 13.74) | 0.45 | 99 |

**Abbreviations:** 2C (two-component), 3C (three-component), 4C (four-component), CDAI (Clinical Disease Activity Score), CI (confidence interval), DAS28-CRP (Disease Activity Score of 28 Joints with CRP).

Supplementary Table S9. Association of baseline GlycA values with baseline (pre-treatment) disease activity, methotrexate only, adjusted for age at recruitment and sex.

| **Outcome measure** | **β-coefficient_adj_ (95% CI)** | **p-value** | **n** |
| --- | --- | --- | --- |
| 4C-DAS28-CRP | 1.25 (0.48 – 2.02) | 2.01E-03 | 99 |
| 3C-DAS28-CRP | 1.13 (0.37 – 1.89) | 4.31E-03 | 99 |
| 2C-DAS28-CRP | 1.97 (1.07 – 2.88) | 4.65E-05 | 99 |
| CDAI | 4.17 (-5.68 – 14.02) | 0.41 | 99 |

**Abbreviations:** 2C (two-component), 3C (three-component), 4C (four-component), CDAI (Clinical Disease Activity Score), CI (confidence interval), DAS28-CRP (Disease Activity Score of 28 Joints with CRP).

Supplementary Table S10. Association of baseline GlycA values with poor treatment response outcomes after 3 months, both drugs combined, univariable analysis.

| **Outcome measure** | **OR (95% CI)** | **p-value** | **n** |
| --- | --- | --- | --- |
| Poor EULAR response (4C-DAS28-CRP) | 0.86 (0.66 – 1.12) | 0.26 | 180 |
| Poor EULAR response (3C-DAS28-CRP) | 0.24 (0.06 – 0.99) | 0.04* | 180 |
| Poor EULAR response (2C-DAS28-CRP) | 0.56 (0.14 – 2.31) | 0.42 | 180 |
| Highly active CDAI (CDAI >22) | 0.68 (0.18 – 2.63) | 0.58 | 168 |

**Abbreviations:** 2C (two-component), 3C (three-component), 4C (four-component), CDAI (Clinical Disease Activity Score), CI (confidence interval), DAS28-CRP (Disease Activity Score of 28 Joints with CRP), EULAR (European Alliance of Associations of Rheumatology), OR (odds ratio).

Supplementary Table S11. Association of baseline GlycA values with poor treatment response outcomes after 3 months, both drugs combined, adjusted for age at recruitment, sex, drug and baseline DAS28-CRP/CDAI.

| **Outcome measure** | **OR_adj_ (95% CI)** | **p-value** | **n** |
| --- | --- | --- | --- |
| Poor EULAR response (4C-DAS28-CRP) | 0.43 (0.09 – 1.99) | 0.28 | 180 |
| Poor EULAR response (3C-DAS28-CRP) | 0.24 (0.05 – 1.22) | 0.08 | 180 |
| Poor EULAR response (2C-DAS28-CRP) | 2.07 (0.37 – 11.49) | 0.40 | 180 |
| Highly active CDAI (CDAI >22) | 0.40 (0.09 – 1.75) | 0.23 | 168 |

**Abbreviations:** 2C (two-component), 3C (three-component), 4C (four-component), CDAI (Clinical Disease Activity Score), CI (confidence interval), DAS28-CRP (Disease Activity Score of 28 Joints with CRP), EULAR (European Alliance of Associations of Rheumatology), OR_adj_ (adjusted odds ratio).

Supplementary Table S12. Association of baseline GlycA values with poor treatment response outcomes after 3 months, adalimumab only, univariable analysis.

| **Outcome measure** | **OR (95% CI)** | **p-value** | **n** |
| --- | --- | --- | --- |
| Poor EULAR response (4C-DAS28-CRP) | 0.52 (0.04 – 7.48) | 0.63 | 83 |
| Poor EULAR response (3C-DAS28-CRP) | 0.19 (0.01 – 3.40) | 0.263 | 83 |
| Poor EULAR response (2C-DAS28-CRP) | 0.69 (0.07 – 6.73) | 0.75 | 83 |
| Highly active CDAI (CDAI >22) | 0.99 (0.14 – 7.15) | 0.99 | 81 |

**Abbreviations:** 2C (two-component), 3C (three-component), 4C (four-component), CDAI (Clinical Disease Activity Score), CI (confidence interval), DAS28-CRP (Disease Activity Score of 28 Joints with CRP), EULAR (European Alliance of Associations of Rheumatology), OR (odds ratio).

Supplementary Table S13. Association of baseline GlycA values with poor treatment response outcomes after 3 months, adalimumab only, adjusted for age at recruitment, sex and baseline DAS28-CRP/CDAI.

| **Outcome measure** | **OR_adj_ (95% CI)** | **p-value** | **n** |
| --- | --- | --- | --- |
| Poor EULAR response (4C-DAS28-CRP) | 0.67 (0.04 – 10.26) | 0.78 | 83 |
| Poor EULAR response (3C-DAS28-CRP) | 0.37 (0.02 – 7.56) | 0.52 | 83 |
| Poor EULAR response (2C-DAS28-CRP) | 1.66 (0.12 – 22.55) | 0.71 | 83 |
| Highly active CDAI (CDAI >22) | 0.72 (0.09 – 6.03) | 0.76 | 81 |

**Abbreviations:** 2C (two-component), 3C (three-component), 4C (four-component), CDAI (Clinical Disease Activity Score), CI (confidence interval), DAS28-CRP (Disease Activity Score of 28 Joints with CRP), EULAR (European Alliance of Associations of Rheumatology), OR_adj_ (adjusted odds ratio).

Supplementary Table S14. Association of baseline GlycA values with poor treatment response outcomes after 3 months, methotrexate only, univariable analysis.

| **Outcome measure** | **OR (95% CI)** | **p-value** | **n** |
| --- | --- | --- | --- |
| Poor EULAR response (4C-DAS28-CRP) | 0.34 (0.06 – 1.91) | 0.22 | 97 |
| Poor EULAR response (3C-DAS28-CRP) | 0.17 (0.03 – 1.06) | 0.06 | 97 |
| Poor EULAR response (2C-DAS28-CRP) | 0.45 (0.07 – 2.89) | 0.40 | 97 |
| Highly active CDAI (CDAI >22) | 0.47 (0.07 – 3.12) | 0.43 | 87 |

**Abbreviations:** 2C (two-component), 3C (three-component), 4C (four-component), CDAI (Clinical Disease Activity Score), CI (confidence interval), DAS28-CRP (Disease Activity Score of 28 Joints with CRP), EULAR (European Alliance of Associations of Rheumatology), OR (odds ratio).

Supplementary Table S15. Association of baseline GlycA values with poor treatment response outcomes after 3 months, methotrexate only, adjusted for age at recruitment, sex and baseline DAS28-CRP/CDAI.

| **Outcome measure** | **OR_adj_ (95% CI)** | **p-value** | **n** |
| --- | --- | --- | --- |
| Poor EULAR response (4C-DAS28-CRP) | 0.36 (0.06 – 2.25) | 0.27 | 97 |
| Poor EULAR response (3C-DAS28-CRP) | 0.20 (0.03 – 1.40) | 0.11 | 97 |
| Poor EULAR response (2C-DAS28-CRP) | 1.55 (0.14 – 17.20) | 0.72 | 97 |
| Highly active CDAI (CDAI >22) | 0.27 (0.03 – 2.10) | 0.21 | 87 |

**Abbreviations:** 2C (two-component), 3C (three-component), 4C (four-component), CDAI (Clinical Disease Activity Score), CI (confidence interval), DAS28-CRP (Disease Activity Score of 28 Joints with CRP), EULAR (European Alliance of Associations of Rheumatology), OR_adj_ (adjusted odds ratio).

Supplementary Table S16. Comparison between patients with RA in remission after three months of treatment (adalimumab or methotrexate) and HCs, using a Mann-Whitney U test to compare groups.

| **Remission definition** | **W** | **p-value** | **n** |
| --- | --- | --- | --- |
| 4C-DAS28-CRP <2.6 | 282 | 0.82 | 35 |
| 3C-DAS28-CRP <2.6 | 333 | 0.49 | 33 |
| 2C-DAS28-CRP <2.6 | 985 | 0.38 | 92 |
| CDAI <2.8 | 62 | 0.57 | 12 |

**Abbreviations:** 2C (two-component), 3C (three-component), 4C (four-component), CDAI (Clinical Disease Activity Score), DAS28-CRP (Disease Activity Score of 28 Joints with CRP).

Supplementary Table S17. Comparison between patients with active RA (not in remission) after three months of treatment (adalimumab or methotrexate) and HCs, using a Mann-Whitney U test to compare groups.

| **Active RA definition** | **W** | **p-value** | **n** |
| --- | --- | --- | --- |
| 4C-DAS28-CRP ≥2.6 | 1299 | 0.01 | 147 |
| 3C-DAS28-CRP ≥2.6 | 1248 | 0.01 | 149 |
| 2C-DAS28-CRP ≥2.6 | 596 | 2.29E-03 | 90 |
| CDAI ≥2.8 | 1519 | 0.02 | 170 |

Supplementary Table S18. Correlation of baseline GlycA values with disease activity scores and their sub-components after 3 months, both drugs combined.

| **Outcome measure** | **r (95% CI)** | **p-value** | **n** |
| --- | --- | --- | --- |
| 4C-DAS28-CRP | 0.03 (-0.12 – 0.17) | 0.73 | 180 |
| 3C-DAS28-CRP | 0.03 (-0.11 – 0.18) | 0.65 | 180 |
| 2C-DAS28-CRP | 0.11 (-0.03 – 0.26) | 0.13 | 180 |
| CDAI | -0.03 (-0.18 – 0.11) | 0.65 | 180 |
| TJC | -0.08 (-0.22 – 0.07) | 0.29 | 180 |
| SJC | 0.00 (-0.14 – 0.15) | 0.98 | 180 |
| Patient GH | -0.01 (-0.15 – 0.14) | 0.94 | 180 |
| Physician GH | 0.05 (-0.10 – 0.20) | 0.50 | 180 |
| CRP | 0.27 (0.12 – 0.40) | 3.09E-04* | 180 |

**Abbreviations:** 2C (two-component), 3C (three-component), 4C (four-component), CDAI (Clinical Disease Activity Index), CRP (C-reactive protein), DAS28-CRP (Disease Activity Score of 28 Joints with CRP), GH (global health using 0 – 100mm visual analogue scale), r (Pearson’s correlation coefficient), SJC (swollen joint count), TJC (tender joint count).

Supplementary Table S19. Correlation of baseline GlycA values with disease activity scores and their sub-components after 3 months, adalimumab only.

| **Outcome measure** | **r (95% CI)** | **p-value** | **n** |
| --- | --- | --- | --- |
| 4C-DAS28-CRP | 0.06 (-0.16 – 0.27) | 0.58 | 83 |
| 3C-DAS28-CRP | 0.10 (-0.12 – 0.31) | 0.37 | 83 |
| 2C-DAS28-CRP | 0.19 (-0.02 – 0.39) | 0.08 | 83 |
| CDAI | -0.00 (-0.22 – 0.21) | 0.98 | 83 |
| TJC | -0.02 (-0.24 – 0.19) | 0.82 | 83 |
| SJC | 0.05 (-0.17 – 0.26) | 0.67 | 83 |
| Patient GH | -0.07 (-0.28 – 0.15) | 0.54 | 83 |
| Physician GH | 0.05 (-0.17 – 0.26) | 0.67 | 83 |
| CRP | 0.36 (0.15 – 0.53) | 9.00E-04* | 83 |

**Abbreviations:** 2C (two-component), 3C (three-component), 4C (four-component), CDAI (Clinical Disease Activity Index), CRP (C-reactive protein), DAS28-CRP (Disease Activity Score of 28 Joints with CRP), GH (global health using 0 – 100mm visual analogue scale), r (Pearson’s correlation coefficient), SJC (swollen joint count), TJC (tender joint count).

Supplementary Table S20. Correlation of baseline GlycA values with disease activity scores and their sub-components after 3 months, methotrexate only.

| **Outcome measure** | **r (95% CI)** | **p-value** | **n** |
| --- | --- | --- | --- |
| 4C-DAS28-CRP | 0.00 (-0.20 – 0.20) | 0.99 | 97 |
| 3C-DAS28-CRP | -0.02 (-0.22 – 0.18) | 0.87 | 97 |
| 2C-DAS28-CRP | 0.04 (-0.16 – 0.24) | 0.69 | 97 |
| CDAI | -0.06 (-0.26 – 0.14) | 0.57 | 97 |
| TJC | -0.13 (-0.32 – 0.08) | 0.22 | 97 |
| SJC | -0.04 (-0.24 – 0.16) | 0.67 | 97 |
| Patient GH | 0.06 (-0.15 – 0.25) | 0.59 | 97 |
| Physician GH | 0.07 (-0.13 – 0.26) | 0.50 | 97 |
| CRP | 0.18 (-0.02 – 0.36) | 0.08 | 97 |

**Abbreviations:** 2C (two-component), 3C (three-component), 4C (four-component), CDAI (Clinical Disease Activity Index), CRP (C-reactive protein), DAS28-CRP (Disease Activity Score of 28 Joints with CRP), GH (global health using 0 – 100mm visual analogue scale), r (Pearson’s correlation coefficient), SJC (swollen joint count), TJC (tender joint count).

Supplementary Table S21. Correlations between change in GlycA between baseline and follow-up and change in disease activity scores and their sub-components between baseline and three months, both drugs combined.

| **Outcome measure** | **r (95% CI)** | **p-value** | **n** |
| --- | --- | --- | --- |
| 4C-DAS28-CRP | -0.03 (-0.18 – 0.11) | 0.65 | 179 |
| 3C-DAS28-CRP | -0.03 (-0.18 – 0.12) | 0.68 | 179 |
| 2C-DAS28-CRP | -0.08 (-0.23 – 0.06) | 0.27 | 179 |
| CDAI | -0.06 (-0.21 – 0.08) | 0.40 | 179 |
| TJC | -0.03 (-0.18 – 0.12) | 0.70 | 179 |
| SJC | -0.13 (-0.28 – 0.01) | 0.07 | 179 |
| Patient GH | -0.03 (-0.18 – 0.12) | 0.70 | 179 |
| Physician GH | 0.01 (-0.13 – 0.16) | 0.85 | 179 |
| CRP | -0.07 (-0.22 – 0.08) | 0.33 | 179 |

**Abbreviations:** 2C (two-component), 3C (three-component), 4C (four-component), CDAI (Clinical Disease Activity Index), CRP (C-reactive protein), DAS28-CRP (Disease Activity Score of 28 Joints with CRP), GH (global health using 0 – 100mm visual analogue scale), r (Pearson’s correlation coefficient), SJC (swollen joint count), TJC (tender joint count).

Supplementary Table S22. Correlations between change in GlycA between baseline and follow-up and change in disease activity scores and their sub-components between baseline and three months, adalimumab only.

| **Outcome measure** | **r (95% CI)** | **p-value** | **n** |
| --- | --- | --- | --- |
| 4C-DAS28-CRP | -0.06 (-0.28 – 0.16) | 0.57 | 83 |
| 3C-DAS28-CRP | -0.03 (-0.24 – 0.19) | 0.82 | 83 |
| 2C-DAS28-CRP | -0.16 (-0.37 – 0.06) | 0.15 | 83 |
| CDAI | -0.09 (-0.30 – 0.13) | 0.44 | 83 |
| TJC | 0.05 (-0.17 – 0.26) | 0.68 | 83 |
| SJC | -0.19 (-0.39 – 0.03) | 0.09 | 83 |
| Patient GH | -0.14 (-0.35 – 0.08) | 0.20 | 83 |
| Physician GH | -0.09 (-0.30 – 0.13) | 0.42 | 83 |
| CRP | -0.10 (-0.31 – 0.12) | 0.36 | 83 |

**Abbreviations:** 2C (two-component), 3C (three-component), 4C (four-component), CDAI (Clinical Disease Activity Index), CRP (C-reactive protein), DAS28-CRP (Disease Activity Score of 28 Joints with CRP), GH (global health using 0 – 100mm visual analogue scale), r (Pearson’s correlation coefficient), SJC (swollen joint count), TJC (tender joint count).

Supplementary Table S23. Correlations between change in GlycA between baseline and follow-up and change in disease activity scores and their sub-components between baseline and three months, methotrexate only.

| **Outcome measure** | **r (95% CI)** | **p-value** | **n** |
| --- | --- | --- | --- |
| 4C-DAS28-CRP | -0.19 (-0.22 – 0.18) | 0.86 | 96 |
| 3C-DAS28-CRP | -0.03 (-0.23 – 0.17) | 0.75 | 96 |
| 2C-DAS28-CRP | 0.00 (-0.20 – 0.20) | 0.99 | 96 |
| CDAI | -0.09 (-0.30 – 0.13) | 0.44 | 96 |
| TJC | -0.08 (-0.28 – 0.12) | 0.43 | 96 |
| SJC | -0.07 (-0.27 – 0.13) | 0.50 | 96 |
| Patient GH | 0.03 (-0.17 – 0.23) | 0.79 | 96 |
| Physician GH | 0.06 (-0.14 – 0.26) | 0.55 | 96 |
| CRP | -0.07 (-0.26 – 0.14) | 0.52 | 96 |

**Abbreviations:** 2C (two-component), 3C (three-component), 4C (four-component), CDAI (Clinical Disease Activity Index), CRP (C-reactive protein), DAS28-CRP (Disease Activity Score of 28 Joints with CRP), GH (global health using 0 – 100mm visual analogue scale), r (Pearson’s correlation coefficient), SJC (swollen joint count), TJC (tender joint count).

**REFERENCES**

1. Hensor EMA, McKeigue P, Ling SF, et al. Validity of a two-component imaging-derived disease activity score for improved assessment of synovitis in early rheumatoid arthritis. *Rheumatology (Oxford)* 2019;58(8):1400-9. doi: 10.1093/rheumatology/kez049 [published Online First: 2019/03/03]

2. DAS-score website [Alternative validated DAS28 formulae]. Available from: <https://www.das-score.nl/en/das-and-das28/how-to-meaure-the-das28/how-to-calculate-the-das28/alternative-validated-formulae> accessed 23rd July 2024.

3. Prevoo ML, van 't Hof MA, Kuper HH, et al. Modified disease activity scores that include twenty-eight-joint counts. Development and validation in a prospective longitudinal study of patients with rheumatoid arthritis. *Arthritis Rheum* 1995;38(1):44-8.

4. Aletaha D, Nell VP, Stamm T, et al. Acute phase reactants add little to composite disease activity indices for rheumatoid arthritis: validation of a clinical activity score. *Arthritis Res Ther* 2005;7(4):R796-806. doi: 10.1186/ar1740

5. van Gestel AM, Prevoo ML, van 't Hof MA, et al. Development and validation of the European League Against Rheumatism response criteria for rheumatoid arthritis. Comparison with the preliminary American College of Rheumatology and the World Health Organization/International League Against Rheumatism Criteria. *Arthritis Rheum* 1996;39(1):34-40.

6. Aletaha D, Martinez-Avila J, Kvien TK, Smolen JS. Definition of treatment response in rheumatoid arthritis based on the simplified and the clinical disease activity index. *Ann Rheum Dis* 2012;71(7):1190-6. doi: 10.1136/annrheumdis-2012-201491 [published Online First: 20120327]
